# Postoperative outcomes and their risk factors in left pancreatectomy with and without multivisceral resection

**DOI:** 10.1038/s41598-025-89251-2

**Published:** 2025-02-14

**Authors:** Maximilian Brunner, Ilvie Loeser, Georg F. Weber, Robert Grützmann, Christian Krautz

**Affiliations:** Department of General and Visceral Surgery, Friedrich-Alexander-University Erlangen-Nürnberg (FAU), Krankenhausstraße 12, 91054 Erlangen, Germany

**Keywords:** Left pancreatectomy, Risk factors, Morbidity, Postoperative pancreatic fistula, Residual pancreatic function., Diseases, Medical research

## Abstract

The aim of the present study was to identify risk factors associated with postoperative morbidity and mortality in patients undergoing isolated left pancreatectomy and those undergoing left pancreatic resection as part of a multivisceral resection. We performed a retrospective analysis of 296 adult patients who underwent elective left pancreatectomy from 2005 to 2022 at the University Hospital Erlangen. Patient demographics, pre- and intraoperative findings, along with postoperative outcomes, were collected and tested as predictive factors for various short-term postoperative parameters. Isolated left pancreatectomy (LP) was performed in 173 patients, while 123 patients underwent left pancreatectomy as part of a multivisceral resection (multivisceral LP). Multivisceral LP was associated with a higher rate of major morbidity (27% vs. 17%, *p* = 0.043) and mortality (7% vs. 2%, *p* = 0.046) compared to LP. Independent risk factors for major morbidity included the need for intraoperative blood transfusion and oncological lymphadenectomy in the LP group and longer operative time in the multivisceral LP group. CR-POPF was associated with the indication for surgery in the LP group. Independent risk factors for re-surgery included intraoperative blood transfusion in the LP group and ASA III or IV in the multivisceral LP group. Cardiovascular diseases were associated with higher mortality in the LP group, while COPD was the only risk factor for mortality in the multivisceral LP group. Multivisceral left pancreatectomy is associated with worse outcomes compared to isolated left pancreatectomy. In both groups, relevant risk factors predict postoperative complications. Patients with these identified risk factors should receive close monitoring during the postoperative course to anticipate outcomes with an increased risk of complications.

## Introduction

After pancreatoduodenectomy in its various forms, left pancreatectomy represents the second most common surgical procedure in pancreatic surgery. Left pancreatectomy is primarily conducted for diverse pancreatic indications, encompassing inflammatory conditions, benign and malignant neoplasms. Additionally, it can be part of multivisceral resections, particularly for extensive extrapancreatic neoplasms originating from the gastric or colonic regions or from sarcomas.

Initially introduced by M. K. Duval in 1954 and further standardized by W.J. Frey and C.G. Child in subsequent years, left pancreatectomy is nowadays a standardized and safe procedure^1–5^. Variations of this operation include especially spleen preservation or splenectomy and the approach used (open vs. minimally invasive)^6–8^.

Nevertheless, morbidity and mortality rates related to left pancreatectomy are not negligible, especially when performed as part of a multivisceral resection^5,9^. A recent analysis of hospital administrative data showed a hospital mortality of 7.3% for left pancreatectomy in Germany and suggests that it is advisable to carry out this procedure in high-volume hospitals^9,10^.

Therefore, understanding the risk factors that may adversely impact surgical and functional outcomes can enhance the quality of treatment by implementing tailored approaches and individualized postoperative measures for high-risk patients. Perioperative morbidity and mortality rates serve as crucial, universally applicable indicators of surgical treatment quality.

The primary objective of the present study was to identify risk factors associated with the development of major morbidity, CR-POPF, need for re-surgery and in-hospital mortality in patients undergoing isolated left pancreatectomy and those undergoing left pancreatic resection as part of a multivisceral resection.

## Methods

This retrospective analysis includes 296 consecutive patients (age ≥ 18 years), who underwent left pancreatectomy for different indications – also when the primary indication was not pancreatic in nature – at the Department of General and Visceral Surgery of the University Hospital of Erlangen between January 2005 and December 2022. Patients with one of the following criteria were excluded: (1) patients with age younger than 18 years; (2) patients undergoing left pancreatectomy due to acute pancreatitis as the indication, given the distinct risk profile associated with such surgeries. Patients were stratified in patients with isolated left pancreatectomy (LP group) and those undergoing left pancreatic resection as part of a multivisceral resection (Multivisceral LP group). Multivisceral resections were defined as the additional resection of the colon, stomach, liver or kidney. Patients with additional resections of the spleen, adrenal gland, or gallbladder were not classified as having undergone multivisceral resections.

Data regarding patient demographics, comorbidities, preoperative therapies, preoperative blood results, surgical details and intraoperative findings as well as postoperative course were collected and analyzed. All pre- and intraoperative parameters were individually assessed for their impact on various postoperative outcome parameters. Risk factors with a p-value less than 0.05 in the univariate analysis were then subjected to multivariate analysis. The primary objective of this study was to identify risk factors associated with in-hospital major morbidity, clinically relevant postoperative pancreatic fistula (CR-POPF), the necessity for re-surgery and in-hospital mortality in patients undergoing left pancreatectomy stratified into isolated LP and mulitvisceral LP.

Morbidity was defined as any deviation from the normal postoperative course and classified using the Clavien-Dindo classification^11^. Morbidity of grade III or higher, according to Clavien-Dindo, was considered major. Postoperative pancreatic fistula (POPF) and postpancreatectomy hemorrhage (PPH) were defined following the criteria established by the International Study Group of Pancreatic Surgery (ISGPS)^12–14^. Clinically relevant POPF (CR-POPF) included POPF of grade B and C, according to the ISGPS definition.

This study was approved by the Ethics Committee of FAU Erlangen (22-165-Br).

### Surgical procedure of left pancreatectomy

All surgeries were carried out by experienced visceral surgeons with extensive practical expertise in pancreatic surgery. Depending on the surgical indication, different approaches were employed. Oncological procedures encompassed a comprehensive oncological lymphadenectomy and splenectomy. Additional vascular resections and multivisceral resections were performed as needed to achieve an R0 situation. In non-oncological indications, splenic preservation was pursued whenever feasible. In the earlier years of the study period, the open approach was the standard in our hospital. After the implementation of minimally-invasive pancreatic surgery in 2016/2017, the choice between surgical approaches (open vs. minimal-invasive) was determined by factors such as tumor size, the necessity of extended resection and the patients’ conditions (e.g., BMI, previous surgeries). The closure of the pancreatic stump was executed at the surgeon’s discretion; however, in open and robotic approaches, sutures were predominantly used, while in laparoscopic procedures, staplers were primarily employed.

### Statistical analysis

Statistical analyses were performed with SPSS Statistic (Version 28.0, IBM). Comparisons of ordinal and metric data were calculated with Mann Whitney U test. For categorical data, the chi-square test was used. Statistical significance was set a *p* < 0.05. Multivariate analysis was performed with in univariate analysis identified risk factors with a p-value of less than 0.05. As cutoffs for metric risk factors the median was used.

## Results

A total of 296 patients, with a median age of 62 years and a 50% female distribution, underwent left pancreatectomy during the study period and were included in the analysis. The most prevalent indications for left pancreatectomy were pancreatic carcinoma (27%), malignant neoplasms not of pancreatic origin (23%), cystic pancreatic lesions (18%), neuroendocrine neoplasms (16%), and chronic pancreatitis (14%). The majority of left pancreatectomies were performed using an open approach (87%) and 10% included vascular resections.

Isolated left pancreatectomy (LP) was performed in 173 patients (58%), while 123 patients (42%) underwent left pancreatectomy as part of a multivisceral resection (multivisceral LP). Regarding patient characteristics, the multivisceral LP group had a higher proportion of male patients (59 vs. 44%, *p* = 0.010), worse ASA scores (*p* = 0.005), a lower BMI (24.7 vs. 26.2 kg/m^2^, *p* = 0.002), a higher rate of neoadjuvant therapy (37 vs. 8%, *p* < 0.001) as well as lower preoperative hemoglobin (12.3 vs. 13.3 g/dl, p = < 0.001) and albumin (41.2 vs. 42.2 g/l, *p* = 0.033) and higher preoperative CRP levels (7 vs. 3 mg/l, *p* < 0.001) (Table 1). Regarding surgical details, the multivisceral LP group was associated with a higher rate of malignant diagnoses (*p* < 0.001), a higher rate of open surgical approach, oncological lymphadenectomy and splenectomy (all *p* < 0.001), a higher rate of additional vascular resection (16 vs. 6%, *p* = 0.004), a greater need for intraoperative blood transfusions (36 vs. 14%, *p* < 0.001) and longer operative times (266 vs. 169 min., *p* < 0.001) (Table 2).

**Table 1:** Patients characteristics.

	LP	Multivisceral LP	*p*
Number	173	123	
Age (years), median (IQR)	61 (22)	63 (18)	0.083
Gender, n (%)			**0.010**
Female	97 (56)	50 (41)	
Male	76 (44)	73 (59)	
ASA, n (%)			**0.005**
I	8 (5)	8 (7)	
II	123 (71)	62 (50)	
III	37 (21)	46 (37)	
IV	3 (2)	3 (2)	
Unknown	2 (1)	4 (3)	
BMI (kg/m^2^), median (IQR)	26.2 (5.2)	24.7 (4.8)	**0.002**
Alcohol history, n (%)	24 (14)	17 (14)	1.000
Smoking history, n (%)	40 (23)	37 (30)	0.182
Comorbidities, n (%)			
Hypertension	85 (49)	61 (50)	1.000
Diabetes	36 (21)	23 (19)	0.662
Cardiovascular	21 (12)	21 (17)	0.241
Chronic renal insufficiency	14 (8)	7 (6)	0.497
Cerebrovascular	8 (5)	2 (2)	0.203
COPD	5 (3)	2 (2)	0.703
Preoperative immunosuppression, n (%)	7 (4)	2 (2)	0.314
Neoadjuvant therapy, n (%)	14 (8)	45 (37)	**< 0.001**
Neoadjuvant chemotherapy			**< 0.001**
Neoadjuvant chemoradiotherapy	10 (6)	21 (17)	**0.002**
Preoperative WBC (10^9^/l), median (IQR)	7.0 (2.6)	7.1 (3.6)	0.848
Preoperative hemoglobin (g/dl), median (IQR)	13.3 (2.5)	12.3 (3.4)	**< 0.001**
Preoperative creatinine (mg/dl), median (IQR)	0.8 (0.2)	0.8 (0.3)	0.973
Preoperative albumin (g/l), median (IQR)	42.2 (4.7)	41.2 (7.5)	**0.033**
Preoperative CRP (mg/l), median (IQR)	3 (7)	7 (23)	**< 0.001**

*ASA* American Society of Anesthesiologists classification, *BMI* Body mass index, *COPD* Chronic obstructive pulmonary disease, *WBC* White blood cells, *CRP* C-reactive protein, *LP* Isolated left pancreatectomy, *Multivisceral LP* Multivisceral left pancreatectomy. Significant values are in bold.

**Table 2:** Surgical details.

	LP (*n* = 173)	Multivisceral LP (*n* = 123)	*p*
Indication for surgery, n (%)			**< 0.001**
Pancreatic carcinoma	42 (24)	38 (31)	
Neuroendocrine neoplasm	35 (20)	12 (10)	
Cystic pancreatic lesion	48 (27)	4 (3)	
Malignant tumor (not of pancreatic origin)	13 (8)	54 (44)	
Chronic pancreatitis	29 (17)	12 (10)	
Other indication	6 (4)	3 (2)	
Surgical approach, n (%)			**< 0.001**
Open	137 (79)	120 (98)	
Minimal-invasive	36 (21)	3 (2)	
Pancreatic stump closure, n (%)			0.899
Suture	118 (68)	85 (69)	
Stapler	55 (32)	38 (31)	
Additional patch plastic, n (%)	35 (20)	27 (22)	0.773
Oncological lymphadenectomy performed, n (%)	84 (49)	103 (84)	**< 0.001**
Additional vascular surgery, n (%)	10 (6)	20 (16)	**0.004**
Splenectomy, n (%)	134 (78)	117 (95)	**< 0.001**
Intraoperative need of blood transfusion, n (%)	25 (14)	44 (36)	**< 0.001**
Operative time (min), median (IQR)	169 (94)	266 (139)	**< 0.001**

*LP* Isolated left pancreatectomy, *Multivisceral LP* Multivisceral left pancreatectomy. Significant values are in bold.

Detailed patient characteristics are provided in Tables 1 and 2 (Tables 1 and 2).

### Postoperative outcome after left pancreatectomy

In 57% of all patients, in-hospital morbidity occurred, with 37% of cases classified as major (21% of all patients). Specific complications included postoperative pancreatic fistula (POPF) in 33% of cases, clinically relevant POPF (CR-POPF) in 10%, postpancreatectomy hemorrhage (PPH) in 2% and wound healing disorder in 3%. Re-surgery was necessary in 9% of all patients. Nine patients (3%) died during the postoperative hospital stay. The mean length of postoperative hospital stay was 14 days. Forty-four patients (15%) required readmission within 90 days.

Patients undergoing multivisceral LP experienced a higher major morbidity rate (27 vs. 17%, *p* = 0.043) and a higher mortality rate (7 vs. 2%, *p* = 0.046) and needed a longer postoperative hospital stay (15 vs. 12 days, *p* = 0.001). Conversely, CR-POPF occurred more often in the isolated LP group ( 13 vs. 5%, *p* = 0.026) (Table 3).

**Table 3:** Postoperative outcome.

	LP (*n* = 173)	Multivisceral LP (*n* = 123)	*p*
In-hospital morbidity, n (%)	94 (54)	74 (60)	0.499
Major morbidity, n (%)	29 (17)	33 (27)	**0.043**
PPH, n (%)	5 (3)	2 (2)	0.703
POPF, n (%)	64 (37)	34 (27)	0.103
CR-POPF, n (%)	22 (13)	6 (5)	**0.026**
Re-operation, n (%)	13 (7)	14 (11)	0.307
Wound healing disorder, n (%)	5 (3)	4 (3)	1.000
In-hospital mortality, n (%)	4 (2)	9 (7)	**0.046**
Length of postoperative stay (days), median (IQR)	12 (9)	15 (11)	**0.001**
Readmission, n (%)	26 (15)	18 (15)	1.000

*PPH* postpancreatectomy heorrhage, *POPF* postoperative pancreatic fistula, *CR-POPF* clinically relevant postoperative pancreatic fistula, *LP *Isolated left pancreatectomy, *Multivisceral LP* Multivisceral left pancreatectomy. Significant values are in bold.

### Risk factors for postoperative outcome in the isolated LP group

Concerning major morbidity, univariate analysis identified four significant associations: ASA III or IV, cardiovascular disease, oncological lymphadenectomy and the need for intraoperative blood transfusion. Among these potential risk factors, only the performance of an oncological lymphadenectomy (OR 2.9 (95% CI 1.1–7.4, *p* = 0.026) and the need for intraoperative blood transfusion (OR 4.2 (95% CI 1.5–11.6), *p* = 0.006) were confirmed as an independent risk factor in multivariate analysis (Table 4).

**Table 4:** Risk factors for major morbidity.

	LP				Multivisceral LP			
	Univariate	Multivariate			Univariate	Multivariate		
	*p*	OR	95% CI	*p*	*p*	OR	95% CI	*p*
ASA III or IV	**0.003**	2.2	0.8–6.2	0.128	0.444	–	–	–
Cardiovascular disease	**0.038**	1.4	0.4–4.8	0.612	0.462	–	–	–
Oncological lymphadenectomy	**0.007**	**2.9**	**1.1–7.4**	**0.026**	0.727	–	–	–
Need of intraoperative blood transfusion	**0.001**	**4.2**	**1.5–11.6**	**0.006**	**0.030**	2.0	0.8–4.6	0.120
Operative time > 169/266 min	0.588	–	–	–	**0.002**	**1.4**	**1.4–8.6**	**0.006**

*OR* odds ratio, *CI* confidence interval, *ASA* American Society of Anesthesiologists classification, *LP* Isolated left pancreatectomy, *Multivisceral LP* Multivisceral left pancreatectomy. Significant values are in bold.

Regarding re-surgery, three parameters had a p-value less than 0.05: ASA III or IV, cardiovascular disease and the need for intraoperative blood transfusion. Multivariate analysis revealed the need for intraoperative blood transfusions (OR 4.3 (95% CI 1.2–15.2), *p* = 0.021) as only independent risk factor (Table 5).

**Table 5:** Risk factors for re-surgery.

	LP				Multivisceral LP			
	Univariate	Multivariate			Univariate	Multivariate		
	*p*	OR	95% CI	*p*	*p*	OR	95% CI	*p*
ASA III or IV	**0.002**	2.2	1.0–14.4	0.058	**0.038**	**4.7**	**1.3–17.7**	**0.021**
Cardiovascular disease	**0.006**	2.1	0.5–8.9	0.311	0.059	–	–	–
Vascular resection	0.760	–	–	–	**0.045**	4.0	1.0–16.3	0.057
Need of intraoperative blood transfusion	**0.002**	**4.3**	**1.2–15.2**	**0.024**	0.244	–	–	–

*OR* odds ratio, *CI* confidence interval, *ASA* American Society of Anesthesiologists classification, *LP* Isolated left pancreatectomy, *Multivisceral LP *Multivisceral left pancreatectomy. Significant values are in bold.

For clinically relevant postoperative pancreatic fistula (CR-POPF) respectively in-hospital mortality, only one parameter was significantly associated in univariate analysis, rendering a multivariate analysis unnecessary. For CR-POPF, the significant factor was the indication for surgery, while for in-hospital mortality, it was cardiovascular disease as comorbidity (Tables 6 and 7).

**Table 6:** Risk factors for CR-POPF.

	LP				Multivisceral LP			
	Univariate	Multivariate			Univariate	Multivariate		
	*p*	OR	95% CI	*p*	*p*	OR	95% CI	*p*
Indication for surgery	**0.003**	*	*	*	0.966	–	–	–

*OR* odds ratio, *CI* confidence interval, *LP* Isolated left pancreatectomy, *Multivisceral LP* Multivisceral left pancreatectomy. *No multiple regression analysis was performed due to only one parameter showing significance in the univariate analysis. Significant values are in bold.

**Table 7:** Risk factors for in-hospital mortality.

	LP				Multivisceral LP			
	Univariate	Multivariate			Univariate	Multivariate		
	*p*	OR	95% CI	*p*	*p*	OR	95% CI	*p*
Cardiovascular disease	**0.045**	*	*	*	0.192	–	–	–
COPD	1.000	–	–	–	**0.005**	*	*	*

*OR* odds ratio, *CI* confidence interval, *LP* Isolated left pancreatectomy, *Multivisceral LP* Multivisceral left pancreatectomy. *No multiple regression analysis was performed due to only one parameter showing significance in the univariate analysis. Significant values are in bold.

### Risk factors for postoperative outcome in the multivisceral LP group

Concerning major morbidity, univariate analysis identified two significant associations: the need for intraoperative blood transfusion and an operative time > 266 min. Among these potential risk factors, only an operative time > 266 min (OR 1.4 (95% CI 1.4–8.6), *p* = 0.006) was confirmed as an independent risk factor in multivariate analysis (Table 4).

Regarding re-surgery, two parameters were significantly associated in univariate analysis: ASA III or IV and the need of a vascular resection. Multivariate analysis revealed an ASA III or IV score (OR 4.7 (95% CI 1.3–17.7), *p* = 0.021) as only independent risk factor (Table 5).

For CR-POPF, no significant risk factors were identified (Table 6). For in-hospital mortality, COPD as comorbidity was the only significant factor, rendering a multivariate analysis unnecessary (Table 7).

## Discussion

Nowadays, left pancreatectomy has evolved into a standardized and safe procedure. However, it still carries associated morbidity and mortality, particularly in cases where it is undertaken as part of a multivisceral resection^5,9,10,15^. This study aims to scrutinize the risks associated with key postoperative outcomes, while identifying their respective risk factors.

In our cohort, major morbidity was 21%, rising significantly to 27% in patients undergoing multivisceral resection. These results align with previously reported rates, which typically range from 20 to 30%^5–7^. Independent risk factors for major morbidity in isolated LP included oncological lymphadenectomy and the need for intraoperative blood transfusion. For multivisceral LP, a longer operative time (> 266 min) was the sole independent risk factor. These findings reflect increased procedural complexity and surgical extent, explaining the association with higher morbidity rates. Our data are consistent with a recent monocentric study by Loos et al. involving over 2000 patients undergoing left pancreatectomy, which identified suspected intraoperative blood loss, procedure extension and longer operative times as independent risk factors^5^. Although Loos et al. also identified ASA III/IV as an independent risk factor, we observed a significant association only in univariate analysis^5^.

Another important surgical outcome parameter is the reoperation rate, which was 9% in our cohort, with higher rates in the multivisceral LP group (11%) compared to isolated LP (7%), though not statistically significant. This is also consistent with the literature, although some studies have reported lower rates^19–21^. The aforementioned recent study by Loos et al. similarly showed a reoperation rate of 9.3%^5^. Regarding this parameter, our analysis identified the need for intraoperative blood transfusion for isolated LP and ASA score of III or IV for multivisceral LP as independent risk factors. This parameter is relatively underexplored in the literature, making comparison with other studies challenging. However, both risk factors identified in our study appear plausible, as they are recognized risk factors in analysis of other surgical procedures.

The clinically relevant pancreatic fistula is the most important and therefore the most extensively studied complication after left pancreatectomy in the literature^16–18^. Compared to the literature, the rate of clinically relevant postoperative pancreatic fistula (CR-POPF) in our cohort appears quite low at 10%. A meta-analysis of over 8000 patients in 2021 showed a CR-POPF rate of 20.4%, consistent with the data from the already mentioned recent analysis by Loos et al., which reported a rate of 23.3%^5,16^. However, some analyses demonstrate even significantly higher rates of up to 48.5%^15^. Regarding risk factors, numerous factors have been described in the literature: the aforementioned meta-analysis by Chong et al., including 43 studies, concluded that smoking and an open surgical approach were significant risk factors for CR-POPF, while the presence of diabetes was a significant protective factor^16^. In the same metaanalysis, many other risk factors such as pancreatic texture and BMI were considered unclear due to the heterogeneity of results^16^. Our results could not confirm the findings of the meta-analysis. Instead, our data identified the indication for surgery as the sole independent risk factor for CR-POPF in isolated LP, consistent with previous findings linking fibrotic pancreatic tissue in chronic pancreatitis or pancreatic carcinoma with lower fistula risk. Conversely, cystic lesions, often associated with fragile pancreatic tissue, pose a higher risk. The lower CR-POPF rate in multivisceral LP may be attributable to the lower prevalence of cystic lesions in this group. Pancreatic texture is also a frequently mentioned risk factor in the literature for the occurrence of CR-POPF, even though this was not conclusively identified as a clear risk factor in the aforementioned meta-analysis due to heterogeneous results^16–18^.

The most critical outcome measure, mortality, stood at 3% in our whole cohort, comprising 2% in isolated LP and 7% in multivisceral LP. These data are comparable with patient cohorts from other specialized pancreatic centers^5,19–21^. Notably, Loos et al. reported a 90-day mortality of 1.6%, Zhou et al. found 1.0% in their analysis of 303 patients and Soreide et al. reported 1.4% in their study of 554 patients^5,19,20^. Rehman et al. documented a 30-day mortality of 2.4% in another retrospective study^21^. An assessment of German hospital administrative data, incorporating over 12,000 patient records, revealed a hospital mortality of 7.3%^9^. The significant higher mortality rate in patients undergoing multivisceral resections is also be described in previous studies^5^. Our identified risk factors included especially comorbidities, especially cardiovascular for isolated LP and COPD for multivisceral LP. Our findings align with those of Loos et al., who also highlighted cardiovascular comorbidities and multivisceral resections or those involving arterial sections as additional risk factors^5^.

The strength of our work lies in providing an overview of the most relevant outcome parameters and their associated risk factors, stratified by procedural complexity. These data can assist in adequately informing patients about potential risks preoperatively. Moreover, as a consequence, the identified risk factors of the surgical outcome parameters can be used to take early preventive measures in patients at risk and to identify complications early on through intensified postoperative monitoring in order to alleviate the extent of the complication.

Several limitations exist regarding our data: First, the retrospective nature of this study and single-center design can incurre some bias and can limit generalizability. Multicenter trials are needed for further validation. Second, stratification reduced the sample sizes of analyzed cohorts, potentially limiting statistical power. Third, most patients underwent open surgery, which may not reflect current minimally invasive standards, particularly for isolated LP. For multivisceral resections, however, open surgery remains the standard. Lastly, functional outcomes, such as endocrine and exocrine insufficiency, were not assessed due to insufficient follow-up data and inconsistent evaluation in our cohort.

## Conclusion

Left pancreatectomy is associated with relevant morbidity and mortality, especially when performed as part of a multivisceral resection. Therefore, precise risk assessment plays a crucial role in identifying those at a higher risk for adverse surgical outcomes. Particularly, modifiable risk factors like intraoperative blood transfusions can be addressed through approaches such as strictly blood-sparing surgeries. Nevertheless, risk classification remains valuable, as individuals at risk may derive benefits from enhanced postoperative attention and more personalized therapeutic approaches.

## Data Availability

All relevant data supporting the conclusions are included within the manuscript and the tables.
